# Taxol^®^: The First Microtubule Stabilizing Agent

**DOI:** 10.3390/ijms18081733

**Published:** 2017-08-09

**Authors:** Chia-Ping Huang Yang, Susan Band Horwitz

**Affiliations:** 1 Department of Molecular Pharmacology, Albert Einstein College of Medicine, Bronx, NY 10461, USA; chia-ping.h.yang@einstein.yu.edu; 2 Department of Obstetrics and Gynecology and Women’s Health, Division of Gynecologic Oncology, Albert Einstein College of Medicine, Bronx, NY 10461, USA

**Keywords:** Taxol^®^, drug binding site, photoaffinity labeling, drug resistance, tubulin isotypes

## Abstract

Taxol^®^, an antitumor drug with significant activity, is the first microtubule stabilizing agent described in the literature. This short review of the mechanism of action of Taxol^®^ emphasizes the research done in the Horwitz’ laboratory. It discusses the contribution of photoaffinity labeled analogues of Taxol^®^ toward our understanding of the binding site of the drug on the microtubule. The importance of hydrogen/deuterium exchange experiments to further our insights into the stabilization of microtubules by Taxol^®^ is addressed. The development of drug resistance, a major problem that arises in the clinic, is discussed. Studies describing differential drug binding to distinct β-tubulin isotypes are presented. Looking forward, it is suggested that the β-tubulin isotype content of a tumor may influence its responses to Taxol^®^.

## 1. Introduction

**Figure 1:**
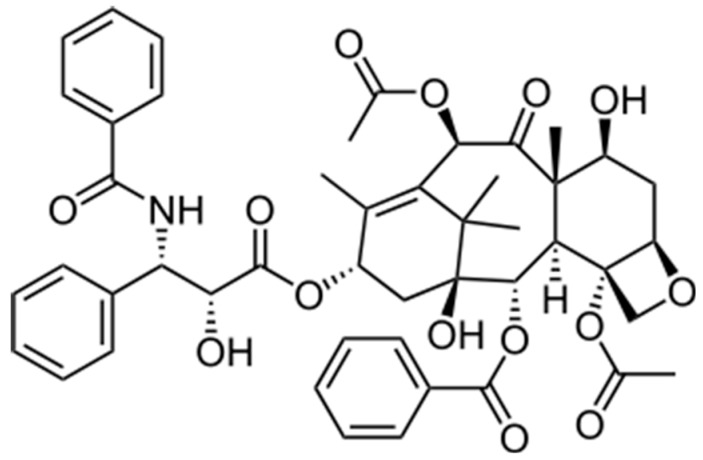
Structure of Taxol^®^.

The stabilization of microtubules by Taxol^®^ (Figure 1), a diterpenoid of natural product origin, was first described in an in vitro microtubule assembly assay in the late 1970s [1] and a year later in mouse fibroblast cells [2]. This represented a novel mechanism of action for a small molecule with the potential to be an important antitumor agent. This short review highlights the contributions of the Horwitz’ Laboratory to our understanding of the mechanism of action of Taxol^®^. 

Taxol^®^ was isolated by Drs. Wall and Wani and their collaborators from the bark of the tree *Taxus brevifolia*, also known as the Western Yew or pacific Yew. They also determined the correct structure of the molecule, not an easy task in the 1960s, and reported in a landmark paper that the compound had antitumor activity in several experimental systems [3]. Taxol^®^ is an architecturally complex molecule whose extreme hydrophobicity has made it a difficult drug to formulate for human use. Due to the limited solubility of the drug, the vehicle used for patients is a mixture of cremophor/ethanol which alone may display some toxic effects. Due to hypersensitivity reactions that occurred with some patients, premedication with corticosteroids and antihistamines were administered. Taxol^®^ has been used in many types of solid tumors, but particularly in breast and ovarian malignancies. The major toxicities caused by Taxol^®^ are neutropenia and peripheral neuropathies [4].

Early studies indicated that the drug was a potent inhibitor of cell replication and migration [2] with the cells being blocked in the late G_2_/M phase of the cell cycle. The drug has the capacity to shift the equilibrium between soluble tubulin and the microtubule polymer in favor of the latter, and thereby reduce the critical concentration of tubulin required to form a microtubule. This ability of the drug to promote microtubule assembly in vitro occurs in the absence of GTP, microtubule-associated proteins, physiological temperatures, and is highly specific to tubulin [5]. Such microtubules are resistant to depolymerization by calcium and cold conditions, which depolymerize normal microtubules [1]. Microtubules have a variety of important functions in eukaryotic cells, being involved in mitosis, maintenance of cell shape, motility and intracellular trafficking of organelles and macromolecules. In order to participate in these activities, microtubules must be highly dynamic and Taxol^®^ has the capacity to inhibit the dynamicity of microtubules [6]. 

Biologically active [^3^H]Taxol^®^ was prepared to probe directly the binding of the drug to tubulin [7]. Experiments indicated that Taxol^®^ binds specifically and reversibly to microtubules with a stoichiometry approaching unity [7]. Such studies indicated that there is a binding site for the drug on the intact microtubule. The idea that Taxol^®^ had a binding site on the microtubule was new and represented a major change from the concept that small, natural product molecules, such as colchicine and the vinca alkaloids, had a binding site on the tubulin dimer and their presence inhibited microtubule assembly [1]. 

One of the observations that was made in cells incubated with Taxol^®^ was the formation of distinct bundles of microtubules in interphase cells [2]. These microtubule bundles are diagnostic of Taxol^®^ treatment and are observed in the white cells of patients being treated with the drug [8]. Little is known about the formation of these unusual microtubule arrays in interphase cells. Even today, close to forty years after they were first described, we do not understand the mechanism by which microtubules form bundles, although it has been noted that depletion of cellular ATP prevents the characteristic Taxol^®^-induced bundle formation [9]. Abnormal microtubule arrays have been described in a variety of systems after Taxol^®^ treatment. For example in trypanosomes, Taxol^®^ inhibits cytokinesis, yet duplication of cellular organelles continues [10]. Studies done with organotypic mouse spinal cord-ganglion cultures indicated that the distribution and organization of organelle systems in dorsal root ganglion cells were altered after incubation with Taxol^®^, and microtubules often were found arrayed along endoplasmic reticulum cisternae [11]. 

## 2. Taxol^®^-Mediated Cell Death Is Concentration Dependent

Although Taxol^®^ was primarily thought of as a drug that acts in the mitotic phase of the cell cycle, it has become clear that Taxol^®^ has effects on microtubules throughout the cell cycle; its presence in a cell has a variety of consequences, many of which occur in interphase cells [12,13]. For example, in primary human vascular endothelial cells, low concentrations of Taxol^®^ suppress microtubule dynamics and inhibit cell migration [14]. Taxol^®^ alters specific intracellular signal transduction events, such as tyrosine phosphorylation of proteins including mitogen-activated protein (MAP) kinases [15,16], activation of Raf-1 kinase [17,18] and phosphorylation of p66Shc [19]. It was suggested that activation of Raf-1 may be essential for drug-induced apoptosis [17,18]. Our laboratory reported that Taxol^®^-induced cell death in human lung carcinoma cells, A549, may result from two different mechanisms that are concentration dependent. At low concentrations (<9 nM), cell death may occur after an aberrant mitosis by a Raf-1 independent pathway, whereas at higher concentrations (≥9 nM) cell death may be the consequence of a mitotic arrest occurring by a Raf-1-dependent cascade [20].

To further explore the molecular mechanisms underlying the action of microtubule-stabilizing agents (MSAs), we have analyzed gene expression profiles in A549 cells following treatment with increasing concentrations of Taxol^®^ or epothilone B, another MSA. Low concentrations (~10 nM) of these drugs induced aberrant mitosis including aneuploidy and asymmetric/multipolar cell division [21]. At drug concentrations that induced G_2_/M arrest (~40–50 nM), cells escaped from a prolonged mitotic arrest without cell division, resulting in tetraploid G_1_ cells (pseudo G_1_ cells). Altered expression of different genes was correlated with mitotic slippage [22]. Poly (ADP-ribose) polymerase (PARP) cleavage, an early indicator of apoptosis, occurred in cells undergoing mitotic slippage and in aneuploid cells resulting from aberrant mitosis. However, cells arrested in mitosis showed minimal PARP cleavage, but had an increased expression of the apoptosis inhibitor gene, survivin [23]. Our results indicated that abnormal mitotic exit was required for cell death induced by a MSA [22]. 

Accurate chromosome segregation is tightly monitored by the key spindle checkpoints, Mad2 and BubR1. They bind and inhibit p55CDC, which is necessary for the activation of the anaphase promoting complex [24,25]. The cell cycle is arrested at metaphase if the checkpoint detects any defects in microtubule-kinetochore attachment or in the tension of the spindles. Since Taxol^®^ suppresses spindle microtubule dynamics [6], it is believed that the drug may alter the tension of the kinetochore microtubules. We have studied cell cycle progression and analyzed the spindle checkpoint proteins of HeLa cells after low and high concentrations of Taxol^®^ treatment. We found that low concentrations (5–10 nM) of Taxol^®^ caused mitotic delay followed by premature dissociation of p55CDC from Mad2 and BubR1, thereby abrogating the spindle checkpoint and leading to aneuploidy. In contrast, high concentrations (20–50 nM) of Taxol^®^ sustained the protein complex formation, resulting in a mitotic block. It was concluded that the induction of cell death and aneuploidy by low concentrations of Taxol^®^ may result from chromosome missegregation caused by spindle checkpoint defects [26].

In addition to targeting mitosis, MSAs have major effects on mitosis-independent cellular events, such as mediating alterations in cell signaling and trafficking. These pathways are dependent on intact and dynamic microtubules and are downstream targets of microtubule stabilizing agents. Many oncoproteins, including p53, BRCA1 and the androgen receptor, have been shown to associate with or traffic on microtubules [27,28,29,30]. Low concentrations of MSAs suppress microtubule dynamics and influence the nuclear accumulation and microtubule-dependent trafficking of some of these proteins. It has been suggested that interfering with the ability of an essential oncoprotein to traffic on microtubules could disrupt its normal function, possibly leading to cell death [12].

However, cells often adapt to the presence of a drug by developing resistance through a variety of different mechanisms that are difficult to overcome.

## 3. Drug Resistance

Effective chemotherapy can be hindered by the acquisition of drug resistance which often occurs in human tumors. A variety of diverse mechanisms underlying drug resistance have been reported [31,32,33]. One type of resistance, multidrug resistance (MDR), has been studied extensively. MDR cells, when selected for resistance to a single hydrophobic drug, develop resistance to a variety of structurally and functionally unrelated lipophilic agents. MDR cells overproduce a plasma membrane glycoprotein (P-glycoprotein) that acts as an ATP-dependent drug efflux pump to maintain drug concentrations below cytotoxic levels. Our laboratory was the first to use Taxol^®^ to develop murine MDR cells [34] and found that these cells overproduced two isoforms of P-glycoprotein [35].

Many MSA-resistant cell lines that lack the expression of P-glycoprotein have been selected in our laboratory. These include: (1) The Taxol^®^-resistant human lung carcinoma cell line A549.T12 that contains an α-tubulin mutation at residue 379 that is near the C-terminus, a site of interaction with microtubule associated proteins. Elevated levels of microtubule destabilizing factors, such as the active non-phosphorylated form of stathmin and the inactive phosphorylated form of MAP4, are increased in these cells [36]. (2) Epothilone B-resistant A549.EpoB40 cells harbor a Gln to Glu mutation at residue 292 that is near the M-loop of β-tubulin [37]. The interaction between mitotic checkpoint proteins CENP-E and BubR1 is diminished in this resistant cell line [38]. (3) K20T, the Taxol^®^-resistant human breast cancer cell line derived from MDA-MB-231 harbors a Glu to Gly mutation at residue 198 in β-tubulin that is near the intradimer interface within the α/β-tubulin heterodimer. It was suggested that βGlu198 is a critical determinant for microtubule stability and Taxol^®^ resistance [39]. (4) A highly epothilone B-resistant cell line, A549.EpoB480, contains a Val to Phe mutation in tubulin at β60 that confers drug dependence [40]. Taxol^®^-dependent cell lines have been shown to have unstable microtubule organizing centers involving a high degree of microtubule detachment from centrosomes. Taxol^®^ is able to inhibit this process, thereby leading to normal cell division [41].

Also selected were MSA-resistant cell lines from human ovarian cancer Hey cells; an epothilone B-resistant cell line, Hey.EpoB8, and an ixabepilone-resistant cell line, Hey.Ixab80. Several MSA-resistant cell lines were compared by 2D-DIGE proteomics and it was found that a variety of cytoskeletal and cytoskeleton-associated proteins, such as galectin-1, 14-3-3σ and phosphorylated stathmin, were differentially expressed in drug-resistant cells [42].

The mutations found in MSA-resistant cell lines have not been detected in human tumors following chemotherapy; however, they provide information that allow us to further understand the structure of tubulin, effects of drugs on microtubule dynamics, and the mechanisms involved in drug resistance and dependence.

## 4. A Binding Site for Taxol^®^ on the Microtubule

It became clear that to understand the biological activity of Taxol^®^ it would be necessary to delineate its binding site on the microtubule. Since Taxol^®^ did not bind covalently to the microtubule, the question arose as to the best methodology to use in order to identify the binding site. In an initial experiment, [^3^H]Taxol^®^ was found to directly photolabel tubulin and the results indicated that the radiolabeled drug bound covalently to the β-subunit of tubulin [43]. Although this was important information, it was clear that the low level of photoincorporation precluded the ability to use [^3^H]Taxol^®^ to define the Taxol^®^ binding site within β-tubulin. It was decided to undertake a major project that involved collaborating with organic chemists willing to prepare Taxol^®^ analogues bearing photoreactive groups at defined positions in the drug. In addition, it was necessary to have a tritium label close to the photoreactive group so the site of incorporation could be accurately determined. It seemed important to undertake these experiments, since it was becoming clear that Taxol^®^ had activity in human malignancies, particularly in drug-refractory ovarian and breast carcinomas [44,45,46,47].

The first analogue studied, 3′-(p-azidobenzamido)taxol, was prepared by Dr. Charles Swindell and found to covalently bind to β-tubulin after UV irradiation. After formic acid cleavage and subsequent protein sequencing and mass analysis, it was determined that the N-terminal 31 amino acids were the major domain of photoincorporation for this analogue [48].

The second photaffinity analogue of Taxol^®^, 2-(m-azidobenzoyl)taxol (2-m-AzTax), was prepared by Dr. David Kingston. After cyanogen bromide and trypsin digestion, radiolabeled peptides were purified by HPLC, followed by amino acid sequencing. A peptide containing amino acid residues 217–231 of β-tubulin was identified as the major photolabeled domain [49].

**Figure 2:**
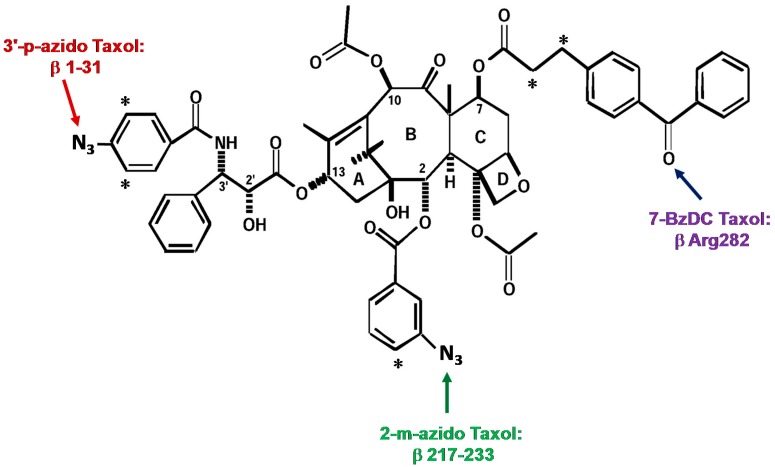
Photolabeling sites on β-tubulin obtained with three photoaffinity analogues of Taxol^®^. Asterisks represent [^3^H].

Whereas the first two photoaffinity Taxol^®^ analogues used were arylazide-containing analogues, the third analogue was a 7-benzophenone analogue of Taxol^®^ prepared by Dr. Iwao Ojima, that allowed us to identify amino acid Arg^282^ in β-tubulin as the site of photoincorporation [50] (Figure 2 for these three photolabeling sites).

In the late 1990s, Nogales and collaborators developed an atomic model of the α, β-tubulin dimer fitted to a 3.7 Å density map by using electron crystallography [51]. Their utilization of zinc-induced tubulin sheets stabilized with Taxotere^®^, a semi-synthetic analogue of Taxol^®^, allowed them to build this model. The binding site for Taxol^®^, as determined by our photoaffinity labeling studies, correlated well with the data obtained with electron crystallography. Our photocrosslinking results allowed us to propose a model of Taxol^®^ binding with microtubules [50] based on the results of Nogales et al. [51]. As noted, the 7-benzophenone analogue of Taxol^®^ photoincorporates into Arg^282^, which is present in the M-loop of β-tubulin [50], part of the Taxol^®^ binding site [51]. The stabilization of microtubules by Taxol^®^ is thought to be the result of the drug’s ability to strengthen the lateral contacts between protofilaments [52]. 

## 5. Insights into the Stabilization of Microtubules by Taxol^®^

The electron crystallography and photoaffinity labeling experiments described above provided information on the binding site for Taxol^®^ and demonstrated that the drug binds in a hydrophobic pocket in β-tubulin. However, there was little information on the effects of Taxol^®^ on the conformation of the entire microtubule. We decided to use hydrogen/deuterium exchange (HDX) coupled to liquid chromatography-electrospray ionization mass spectrometry to examine this question, and we noted that there was a definite reduction in the global deuterium incorporation in both α- and β-tubulin in the presence of Taxol^®^ [53]. Our studies demonstrated that this methodology could be used to study relatively small and specific alterations in the conformation of the microtubule by a drug that binds to the polymer. 

By the 1990s it was clear that Taxol^®^ was an important antitumor drug having been FDA approved for the treatment of ovarian, breast and lung cancers [4]. Today the drug has been given to over one million patients. Due to its success in the clinic and financially, scientists have searched for new agents with a similar mechanism of action. At the same time, it was known that there were problems with Taxol^®^: its aqueous insolubility, the development of drug resistance, and the presence of drug toxicities. Among these new molecules discovered, the following five have been studied in our laboratory by HDX: epothilone B, ixabepilone, discodermolide, peloruside A, and laulimalide [54]. One of the compounds that was of particular interest was discodermolide because it was more water soluble than Taxol^®^ and also was not cross-resistant to Taxol^®^-resistant cell lines that were generated in our laboratory [55]. There was also evidence that the two drugs, Taxol^®^ and discodermolide, acted synergistically in cells and in an ovarian xenograft tumor model in nude mice [55,56]. Our HDX experiments with discodermolide confirmed that the drug binds in the Taxol^®^ binding site in β-tubulin, but interestingly, modeling studies suggested that in contrast to Taxol^®^, discodermolide orients itself away from the M-loop and toward the N-terminal H1-S2 loop of tubulin. These complementary stabilizing effects of Taxol^®^ and discodermolide on the microtubule may explain some of the synergy observed between these two drugs [55,56].

One piece of information revealed by these HDX experiments, that was not appreciated at the time, was the major protection of deuterium exchange observed with both drugs at peptide 212–230. In the next section it becomes clear that this peptide, which represents the leucine cluster region, may be involved in isotype specific drug binding [57]. 

## 6. Taxol^®^ Analogue, 2-(m-Azidobenzoyl)Taxol, Binds Differentially to Distinct β-Tubulin Isotypes

There are eight α- and eight β-tubulin isotypes present in distinct quantities in different human cells, each isotype being the product of an individual gene. In addition to genetic variation, tubulin isotypes are extensively post translationally modified (PTM). Altered expression of β-tubulin isotypes, in particular βIII-tubulin, has been reported in cancer cell lines resistant to MSAs [42,58] including Taxol^®^ [59,60]. βIII-tubulin-mediated Taxol^®^ resistance has been associated with reduced effects on microtubule dynamic instability [61]. It has also been demonstrated that βIII-tubulin counteracts Taxol^®^’s inhibition of cell migration [62]. The relative binding affinities of MSAs have been analyzed by studying their inhibition of [^3^H]2-m-AzTax photoaffinity labeling of tubulins containing distinct β-tubulin isotype content. The results indicated that the inhibitory effects caused by MSAs on photolabeling were very different for β-tubulin from bovine brain (BBT) and chicken erythrocyte (CET) [57]. BBT contains 3% βI-, 58% βII-, 25% βIII- and 13% βIV-tubulin [63] whereas CET contains primarily βVI-tubulin [64,65]. Taxol^®^ had a minimal inhibitory effect on 2-m-AzTax photolabeling of BBT, but a strong inhibitory effect on CET [57]. Discodermolide exhibited opposite effects on photolabeling compared to those of Taxol^®^. In contrast, laulimalide and peloruside A both have stimulatory effects on β-tubulin photolabeling [57]. This latter results are consistent with the finding that these two drugs allosterically stabilize the taxane binding site in the M-loop that establishes lateral tubulin contacts in MTs [66]. 

The covalent binding of 2-m-AzTax to tubulin after UV irradiation allowed us to measure the amount of this Taxol^®^ analogue that binds to each β-tubulin isotype. After photolableing of BBT, we separated tubulin isotypes by high resolution isoelectrofocusing (IEF), followed by cyanogen bromide digestion and mass spectrometry to identify each band on the IEF gel. It was observed that βIII-tubulin binds the least amount of 2-m-AzTax compared to other β-tubulin isotypes [57].

**Figure 3:**
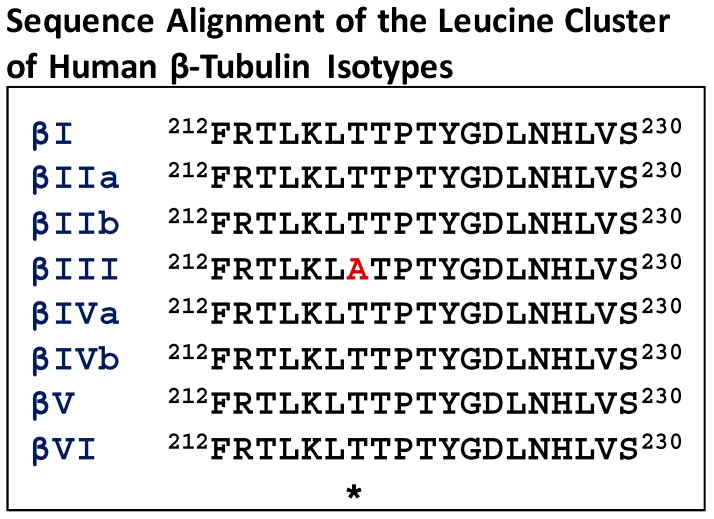
Sequence alignment of the leucine cluster of human β-tubulin isotypes. The asterisk denotes an altered residue (in red).

Many studies have focused on the M-loop residues (β270–β286) which are part of the drug binding pocket. Further analysis of the sequences of β-tubulin near the Taxol^®^ binding site indicated that the leucine cluster region of βIII-tubulin (residues 212–230) contains a unique residue, alanine, at 218, compared to other isotypes that contain threonine (Figure 3). This unique residue is present in human, murine and bovine βIII-tubulin. Based on our photoaffinity labeling and HDX experiments, it became evident that the leucine cluster in β-tubulin was involved in Taxol^®^ binding [49,54,67]. Studies from other laboratories demonstrated that the leucine cluster was involved in Taxol^®^ resistance [68,69].

**Figure 4:**
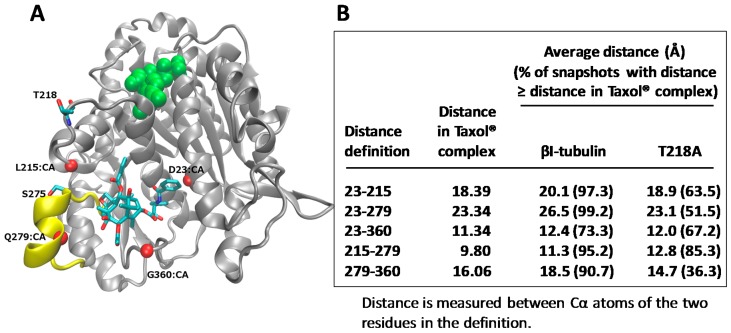
βIII-tubulin subunit contains a unique residue 218 (T218A) in the leucine cluster. (**A**) β-tubulin subunit (grey) in complex with Taxol^®^ (stick representation) and GDP (green). Four C-α (CA) atoms (red spheres) are used to define the binding pocket dimensions. T218 has negligible interactions with Taxol^®^; (**B**) Data from molecular dynamics simulations are shown. Figure adapted from Yang et al. [57].

Molecular models for human βI- and βIII-tubulin in complex with Taxol^®^ were built, and molecular dynamic simulations carried out. The studies indicated that the frequency of Taxol^®^-accommodating conformations decreased significantly in the T218A variant, compared to βI-tubulin (Figure 4). Therefore, it was suggested that the presence of the unique residue, Ala218 in βIII-tubulin, may be a key reason why drug binding to βIII-tubulin is inhibited. Several investigators have considered βIII-tubulin as a drug target for overcoming drug resistance and have designed drugs based on computational modeling of the Taxol^®^ binding site of βIII-tubulin, with the focus on the M-loop [70,71,72]. In the future, the T218A variant should be considered when designing drugs for the binding site in βIII-tubulin. These results link drug response with the β-tubulin isotypes present in a tumor and emphasize the importance of designing Taxol^®^ analogues that specifically interact with βIII-tubulin. A further understanding of the isotypes in human tumors and their response to Taxol^®^ could result in improved therapeutic use of the drug.

## 7. Conclusions

It is clear that Taxol^®^ is part of a large group of drugs of natural product origin that have proven useful in the treatment of disease. The drug has been given to over a million cancer patients and also has shown promise in the treatment of neurodegenerative disease [73]. Taxol^®^ has been used in the preparation of stents for cardiac disease [74,75]. It has proven to be an indispensable tool for studies in cell biology that focus on the role of the tubulin/microtubule system in cellular functions, and also for scientists interested in microtubule structure and dynamics. Natural product chemists, biologists and pharmaceutical companies should continue to search for new natural products with unique chemical structures that may have meaningful medical applications.
